# Evaluation of Physical–Chemical Properties of Contemporary CAD/CAM Materials with Chromatic Transition “Multicolor”

**DOI:** 10.3390/ma16114189

**Published:** 2023-06-05

**Authors:** Monika Lukomska-Szymanska, Mateusz Radwanski, Naji Kharouf, Davide Mancino, Herve Tassery, Corrado Caporossi, Francesco Inchingolo, Aline de Almeida Neves, Yu Fu Chou, Salvatore Sauro

**Affiliations:** 1 Department of General Dentistry, Medical University of Lodz, 251 Pomorska Str., 92-213 Lodz, Poland; 2 Department of Endodontics, Medical University of Lodz, 251 Pomorska Str., 92-213 Lodz, Poland; mateusz.radwanski@umed.lodz.pl; 3 Department of Endodontics and Conservative Dentistry, Faculty of Dental Medicine, Strasbourg University, 67000 Strasbourg, France; dentistenajikharouf@gmail.com (N.K.); mancino@unistra.fr (D.M.); 4 Department of Biomaterials and Bioengineering, INSERM UMR_S 1121, Strasbourg University, 67000 Strasbourg, France; 5 Ecole de Médecine Dentaire de Marseille, Université d’Aix-Marseille, 13385 Marseille, France; herve.tassery@gmail.com; 6 Laboratoire Bioinginierie et Nanoscience, LBN, Université de Montpellier, 545 Pr JL Viala, 34090 Montpellier, France; 7 Independent Researcher, Roma, 65B, 00030 Labico, Italy; c.caporossi1@alice.it; 8 Department of Interdisciplinary Medicine, University of Bari “Aldo Moro”, 70124 Bari, Italy; francesco.inchingolo@uniba.it; 9 Department of Pediatric Dentistry and Orthodontics, Universidade Federal do Rio de Janeiro, Rio de Janeiro 21941-853, Brazil; aline.neves@odonto.ufrj.br; 10 Dental Biomaterials and Minimally Invasive Dentistry, Departamento de Odontología, Facultad de Ciencias de la Salud, Universidad CEU-Cardenal Herrera, C/Del Pozo s/n, Alfara del Patriarca, 46115 Valencia, Spain; yu.chou2@alumnos.uchceu.es

**Keywords:** CAD/CAM, flexural strength, multicolor composites, roughness, ultimate tensile strength, water sorption, SEM

## Abstract

The use of materials for computer-aided design/computer-aided manufacturing (CAD/CAM) has been rapidly increasing in daily practice. However, one of the main issues regarding modern CAD/CAM materials is their aging in the oral environment, which may lead to significant changes in their overall properties. The aim of this study was to compare the flexural strength, water sorption, cross-link density (softening ratio%), surface roughness, and SEM analysis of three modern CAD/CAM “multicolor” composites. Grandio (Grandio disc multicolor—VOCO GmbH, Cuxhaven, Germany), Shofu (Shofu Block HC—Shofu Inc., Kyoto, Japan), and Vita (Vita Enamic multiColor—Vita Zahnfabrik, Bad Sackingen, Germany) were tested in this study. They were prepared in stick-shaped specimens and submitted to different tests after several aging protocols, such as thermocycling and mechanical cycle loading challenge. Further disc-shaped specimens were also created and tested for water sorption, cross-link density, surface roughness, and SEM ultramorphology, before and after storage in an ethanol-based solution. For flexural strength and ultimate tensile strength, Grandio showed the greatest values both at baseline and after aging (*p* < 0.05). Grandio and Vita Enamic presented the highest modulus of elasticity and the lowest water sorption (*p* < 0.05). A significant reduction (*p* < 0.05) in microhardness after ethanol storage (softening ratio%) was observed especially in Shofu. Grandio had the lowest roughness parameters compared to the other tested CAD/CAM materials, while ethanol storage significantly increased the Ra and RSm values in Shofu (*p* < 0.05). Despite the comparable modulus of elasticity of Vita and Grandio, this latter showed greater flexural strength and ultimate tensile strength both at baseline and after aging. Hence, Grandio and Vita Enamic may be employed for the anterior teeth and for those restorations requiring load-bearing capacity. Conversely, aging seems to affect several properties of Shofu, so its use for permanent restorations should be well-pondered based on the clinical situation.

## 1. Introduction

Computer-aided design/computer-aided manufacturing (CAD/CAM) systems have revolutionized the clinical approach and the restorative concepts in prosthodontics. Indeed, there has been a progressive expansion of such a methodology, as well as a continuous innovation in CAD/CAM restorative materials [1]. Nowadays, a wide range of polymer-based, prefabricated CAD/CAM materials is currently available on the market for both temporary and permanent dental restorations.

Ceramics (glass-ceramics, polycrystalline alumina, and zirconium oxide) or resin-based composites can be used to fabricate aesthetic restorations through CAD/CAM technology [2,3,4].

CAD/CAM composite materials, when compared to those in ceramics, may usually exhibit lower aesthetic properties, especially over a long period of service in the oral cavity [5]. The hardness and modulus of elasticity of some CAD/CAM ceramics may resemble those of enamel (1.1–4.9 GPa and 50–120 GPa, respectively), while those of some CAD/CAM composites may be comparable to those of dentin (0.3–0.7 GPa and 10–40 GPa, respectively) [6]. Indeed, it has been reported that restorations of posterior teeth performed with CAD/CAM composites cause less abnormal tooth wear; they represent the main choice for the restoration of teeth in patients affected by bruxism [7,8]. Glass-ceramics, as well as glass-free-ceramics, exhibit values of the flexural modulus (Ef) and Vickers hardness that are considerably higher than those of resin composites (≥60 GPa, >4 GPa versus 9–20 GPa, 0.4 GPa, respectively) [9]. Moreover, CAD/CAM resin composites offer advantages over glass-ceramic blocks, including enhanced margin adaptation and easier fabrication (less brittle) and repair [2,10]. Conversely, CAD/CAM ceramics present greater biocompatibility, lower plaque adherence, and better colour stability compared to CAD/CAM composites [5,11]. Potential discoloration is sometimes a limiting factor when using some resin-based materials, especially for the restoration of anterior teeth [8].

A further category of CAD/CAM materials is represented by resin-ceramic materials, which are characterized by a combination of excellent aesthetic and mechanical properties, reduced brittleness, and increased resistance to fracture [12,13,14,15]. Currently, such materials are manufactured through a specific process based on high pressure and temperature, by which resin is enriched with ceramic filler (>80 wt%). Moreover, there is also a polymer-infiltrated-ceramic network (PICN), in which a ceramic base (>85 wt%) is infiltrated with resin monomers (e.g., UDMA, TEGDMA) (14 wt%) [15]. This latter manufacturing process improves the mechanical properties of such materials, as well as the surface properties, which allows less biofilm formation compared to conventional resin-based composites used for direct restorations [16]. Resin-ceramic materials exhibit high flexural strength, so they have been advocated both for CAD/CAM indirect restorations and in case of reduced thicknesses [2,15,17].

Unfortunately, resin-based materials can absorb water over time, which may lead to swelling and plasticization of the resin matrix. Such a critical condition may induce hydrolytic degradation reduction with consequent reduction of the mechanical properties, especially at the siloxane bonding interface between the resin matrix and fillers [13,18]. Water absorption is exacerbated in the presence of voids, by defects between the polymer chains, and by the degree of hydrophilicity of the polymer matrix [19]. However, it has been widely demonstrated that the process of polymerization used for composite materials in CAD/CAM technology increases the cross-linking density of the polymer network, reducing the number of pores and voids [4,13,20]. Interestingly, the elution of monomers from CAD/CAM resin ceramic materials is still a matter of concern [21].

Nevertheless, the most relevant issue concerning composite materials is still related to the longevity and stability of their overall properties during a long clinical service. Therefore, such an aging scenario needs to be simulated in vitro to understand and predict the aging of materials in vivo. Indeed, several artificial aging protocols based on thermocycling and cyclic load are currently employed in research to simulate the effects of the oral environment on the bulk properties [22]. Moreover, an ethanol-based aging protocol can be employed to challenge the surface properties of restorative materials [23].

Recently, a new class of CAD/CAM materials with chromatic transition known as a multicolor system have been commercialized. For instance, Vita Enamic multiColor is a resin-ceramic material that has been claimed to have an integrated natural colour gradient in six finely nuanced layers, from neck to incisal. The manufacturer indicated that such a material can be used for reconstructions with reduced wall thicknesses that preserve natural tooth structure, posterior crowns that offer load capacity in cases with limited space availability, precise repair of small defects (e.g., indirect cervical fillings/delicate inlays), and non-/minimally invasive reconstruction of occlusal surfaces. Grandio disc multicolor is a composite that has been claimed to imitate the colour gradient of natural teeth from the incisal edge to the tooth neck, with just three layers [24]. The manufacturer affirms that it can be used for the construction of single-tooth restorations such as crowns, inlays, onlays, veneers, and implant-retained crowns, and, due to its special composition, this composite can reflect and absorb light to create the particularly intense chameleon effect, which allows the individual layers of the restoration to transition into each other and produces a highly aesthetic colour gradient. Shofu Block HC (two-layered block) is a resin-based restorative material for use with dental CAD/CAM milling systems. It has been reported to have tooth-like light diffusion and excellent mechanical properties, so that it can be employed for the production of highly aesthetic and long-lasting anterior and posterior restorations such as crowns, inlays, onlays, veneers, and implant-retained crowns.

Unfortunately, these modern materials, in particular the new Grandio disc multicolor, have been recently commercialized, so there is no information available in the literature about their physical–chemical properties when submitted to different aging protocols.

Thus, the aim of this study was to compare some specific physical–chemical properties, including ultimate tensile strength, flexural strength, water sorption, cross-link density, surface roughness, and SEM analysis of three representative multicolour CAD/CAM materials. The null hypothesis was that there would be no differences between the evaluated materials regarding the tested parameters.

## 2. Materials and Methods

The materials used in this study are presented in Table 1.

### 2.1. Ultimate Tensile Strength (UTS), Flexural Strength (FS), and Modulus of Elasticity

Six cubic-shaped specimens (10 mm length × 10 mm height × 7 mm width) were obtained from each CAD/CAM composite tested in this study system using a diamond blade mounted on a low-speed microtome (Remet evolution, REMET, Bologna, Italy). Half of the specimens (n = 3) were submitted to mechanical cycle loading (350,000 cycles; 3 Hz; 70 N). Subsequently, each specimen was cut into sticks (0.9 × 0.9 mm) using a diamond blade mounted on a low-speed microtome (REMET, Bologna, Italy) and 50 sticks were selected from the centre of each specimen and tested for their ultimate tensile strength. The exact cross-sectional area of each tested specimen was measured after failure using a digital calliper. The sticks were glued to a metal jig using a cyanoacrylate gel (ROCKET Heavy DVA; Corona, CA, USA) and stressed to failure in the universal testing machine (Zwick Z005, ZwickRoell GmbH & Co. KG, Ulm, Germany) with a 5 KN load cell and a cross-head speed of 0.5 mm/min. The UTS results were calculated by dividing the failure load values by the surface area and expressed in MPa, and each stick-value was considered as a statistical unit.

Additional blocks of each tested material (n = 6/group) were used to create specimen sticks in accordance with ISO 6872 (4.0 mm in width × 1.2 mm thickness × 25.0 mm length) for ceramic materials, but due to the length of the CAD/CAM blocks a modified length was employed (4.0 mm in width × 1.2 mm thickness × 17.0 mm length) in this study. A total of 30 sticks specimens was prepared for each tested material (30 × 3 different composites = 90 total). They were randomly divided into two sub-groups and (n = 15) immediately tested or (n = 15) submitted to thermocycling aging (5000 cycles, 5–55 °C) in distilled water. All specimens were tested using the universal testing machine (Zwick) equipped with a 3-point bending system. The modulus of elasticity and maximum failure load (Newton) for each specimen were recorded and expressed in MPa. The flexural strength (FS) values (σf, MPa) were calculated by the software of the universal testing machine (TestXpert^®^ III, Zwick) based on the equations as described previously [25,26].

### 2.2. Water Sorption

Ten disc-shaped specimens for each material group (8 mm diameter and 1 mm thickness) were polished using abrasive silicon-carbide (SiC) papers up to 4000-grits under continuous water irrigation and subsequently stored in a silica-containing desiccator at 37 °C. These specimens were used to assess water sorption (WS) according to the ISO 4049/2000 [26]. All the specimens were assessed, and the first baseline values were obtained when a constant mass was achieved (M1). The volume (V) of the specimens (mm^3^) was calculated by measuring the thickness and diameter using a digital calliper (Mitutoyo, Tokyo, Japan). Subsequently, all the specimens were immersed in 5 mL of distilled water at 37 °C and weighed after 7 days, so the second values were recorded (M2). Subsequently, the specimens were dried again in the desiccator and weighed daily until a final constant mass was obtained (M3). WS was calculated using the following Equation (1):

WS = (M2 − M3)/V(1)

### 2.3. Cross-Link Density (CLD) and Surface Roughness

Eight discs-shaped specimens for each material group (8 mm diameter, 2 mm thickness) were polished using abrasive silicon-carbide (SiC) papers up to 4000-grits under continuous water irrigation and subsequently stored in a silica-containing desiccator at 37 °C. The specimens were submitted to Knoop hardness evaluation (Shimadzu HM, Shimadzu Corporation, Kyoto, Japan) before (KH1) after aging (immersion in absolute ethanol at 37 °C for 48 h) to obtain the KH2 value; these values were used to indirectly evaluate CLD [27]. The materials were tested at 10 gf and with a dwell time of 10 s. The Knoop micro-hardness values (KHN) were recorded as the average of ten indentations per specimen (five indentations on the top surface and five on the bottom one). The CLD% was determined using the following Equation (2):

100 − (KHN2/KHN1 × 100)(2)

The surface roughness (Ra, RMS, and RzDIN parameters) of the specimens (n = 5/group) before and after ethanol aging was measured using a profilometer (Surtronic 3+, Taylor Hobson, Leicester, UK). A distance of 1.75 mm was measured in three randomly selected line scans perpendicular to the expected grinding grooves using a standard diamond tip (tip radius 2 μm, tip angle 90°) and a cut-off level of 0.8 (n = 5) and standardization DIN [27].

### 2.4. Scanning Electron Microscopy

Three further discs-shaped specimens for each material group (8 mm diameter, 2 mm thickness) were polished using abrasive silicon-carbide (SiC) papers up to 4000-grits under continuous water irrigation and subsequently stored in a silica-containing desiccator at 37 °C. Half of these specimens were analyzed at 24 h, while the remaining half of the specimens were first immersed in absolute ethanol at 37 °C for 48 h. The specimens were desiccated overnight in an incubator containing absorbent silica and subsequently mounted on aluminium stubs with carbon cement and finally gold-sputter-coated to be analyzed using an ultra-high-resolution analytical focused ion beam scanning electron microscope (FIB-SEM, Thermo Scientific Scios 2 DualBeam, Waltham, MA, USA) in secondary electron mode.

### 2.5. Statistical Analysis

For modulus of elasticity, ultimate flexural strength, ultimate tensile strength, microhardness, and roughness data, the Shapiro–Wilk test disclosed that the data followed a normal distribution; therefore, 2-factor ANOVA repeated measures followed by a Tukey post-hoc test were used to evaluate the effect of aging and the interactions between each composite block. Sorption values were compared by one-way ANOVA (Welch) followed by a Games–Howell post-hoc test (heterogeneous variance correction).

## 3. Results

### 3.1. Ultimate Tensile Strength Test (UTS), Flexural Strength (FS), and Modulus of Elasticity

Grandio and Vita presented the highest elastic modulus both at baseline and after aging (*p* < 0.05). Regarding the tests for FS and UTS, Grandio showed the greatest results both at baseline and after aging (*p* < 0.05). The FS of Shofu and Vita was comparable (*p* > 0.05) both at baseline and after aging. The UTS after aging of Shofu was significantly lower (*p* <0.05) than that of Vita. A significant reduction (*p* < 0.05) of the UTS after aging (5000 cycles, 5–55 °C) was only observed in the specimens created using Shofu (Table 2).

### 3.2. Water Sorption

Grandio and Vita presented the lowest *(p* < 0.05) water sorption values (5.7 and 5.4 µg/mm^3^, respectively), while Shofu presented 22.8 µg/mm^3^ of water sorption (Table 3).

### 3.3. Cross-Link Density (CLD) and Surface Roughness

Vita presented the highest surface hardness before and after ethanol storage *(p* < 0.05), and the lowest (*p* < 0.05) softening ratio (1.56%), with no significant changes after aging (*p* > 0.05). Grandio presented higher (*p* < 0.05) surface hardness before and after ethanol storage compared to Shofu; however, both composites showed a significant reduction in microhardness after aging (*p* < 0.05). However, a lower softening ratio was attained with Grandio and Vita (6.11% and 1.56, respectively), compared to Shofu, which presented a softening ration of 25.45% (Table 4).

Grandio presented the lowest (*p* < 0.05) roughness for all the parameters evaluated in this study (Ra, RSm, and RzDin) at both baseline and after aging, compared to Shofu and Vita. Storage in ethanol (aging treatment) increased Ra and RSm values for Shofu (Table 5).

### 3.4. Scanning Electron Microscopy

**Figure 1:**
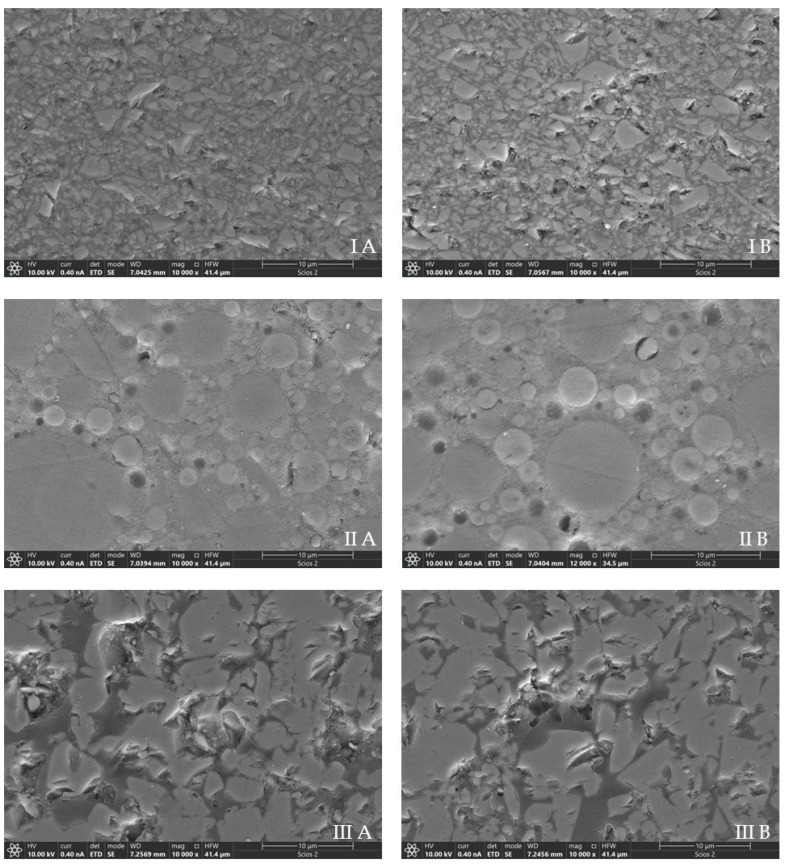
Scanning electron microscope (SEM) images of tested materials before (**A**) and after aging (**B**). **I**—Grandio disc multicolor, **II**—Shofu Block HC, **III**—Vita Enamic multiColor. No evident increase in morphological irregularities can be observed on the surfaces of Grandio and Vita before and after ethanol aging. Conversely, the specimens of Shofu after ethanol aging present more voids due to the loss of the spherical fillers.

The SEM results are depicted in Figure 1. Overall, it was possible to observe no evident morphological differences between the specimens of Grandio and Vita before and after ethanol storage. Conversely, the specimens of Shofu submitted to ethanol aging were characterized by a slight increase in the presence of voids due to the loss of the spherical fillers (Figure 1(IIA)), compared to the control specimens, which were only polished and immediately observed in SEM (Figure 1(IIA)).

## 4. Discussion

In modern dentistry, it is important to choose a restorative material with the most appropriate mechanical and optical/aesthetic properties to satisfy the patient’s needs [28]. Although ceramics may offer superior aesthetic properties, especially when restoring anterior teeth, the use of composite materials is currently very popular thanks to their relatively low cost and to the fact that they are ease to process and to repair intraorally [5].

Dental restorations once placed in the oral environment are subjected to stress-related fractures, dissolution, and disintegration caused by constant changes in temperature and pH (e.g., food, drinks, and acids produced by bacteria) [29,30]. Moreover, some restorative materials may wear out over time, and this may contribute to the reduction in their initial mechanical and aesthetic properties [15,31,32,33].

In a clinical scenario, restorative materials are exposed during mastication to continuous flexural and compressive stresses; it is therefore important that dental materials can exhibit proper flexural and tensile properties to withstand possible fractures [34]. The evaluation of the flexural and tensile strength are important tests that offer an indication of the overall mechanical properties of a specific material; the higher the values, the greater the asset and the longevity of that material [5]. According to the ISO 4049 standard, materials used for occlusal surfaces’ replacement must have a three-point bending strength equal to or greater than 80 MPa [35]. However, it is recommended that all materials claimed for dental reconstructions should have at least a flexural strength higher than 50 MPa [35].

The null hypothesis tested in this study was rejected because there were several significant differences between the evaluated parameters of the tested CAD/CAM materials both initially and after aging. Indeed, the highest FS values was observed in Grandio (324.7 MPa), followed by Shofu (164.3 MPa) and Vita (161.1 MPa), and the FS values were in accordance with those provided by the manufacturers (Grandio 330 MPa, Shofu 170–190 MPa, Vita 150–160 MPa) [7,15,36]. Shofu exhibited significantly greater FS values than Vita, but these results are in contrast to the results reported in a previous study [34], where Shofu showed comparable FS values to Vita. On the other hand, the FS values of Vita Enamic were comparable to those reported in previous studies where lithium di-silicate ceramics were also tested [15,37,38].

Shofu and Vita specimens, at baseline and after aging, presented flexural strength values comparable to those of sound dentine (212.9 ± 41.9 MPa), while Grandio exceeded such values. In addition, all evaluated materials exceeded the FS of enamel (60–90 MPa) [39].

Moreover, the effect of material aging (thermocycling and media storage) on the FS values is still under dispute [40,41,42]. This process may simulate the physiological aging of the material and induces temperature changes at the oral cavity environment [43,44]. In the present study, 5000 thermocycles were applied, which may theoretically correspond to a more than 6-month clinical service; it is the minimum requirement to challenge the longevity of restorative materials in vitro [45].

The storage in ethanol resulted in no significant reduction in the FS of specimens created with Grandio (3.8%) [46]. It has been reported in previous investigations that ethanol storage can reduce the mechanical properties of CAD/CAM composites, including hardness and flexural strength [47,48]. This latter situation is due to the fact that ethanol can penetrate the resin matrix, causing disintegration of bonds between the filler and the resin matrix [49]; this is exactly what the current study confirmed by the SEM result obtained with the specimens of Shofu. In addition, it has been demonstrated that CAD/CAM composites immersed in ethanol may elute a significant amount of components and they can be affected by the formation of voids, which contribute to the reduction in the durability of such materials in vivo [47,50]. In the current study, the thermocycling aging induced no significant changes in the FS of the tested materials. Conversely, some previous studies reported a reduction of the FS by 20% after aging of Vita Enamic in artificial saliva [32,51,52], and a similar decrease in the FS for Shofu HC block (20.06%) after 3 months of storage in water [41]. On the other hand, our results are in accordance with those of Egilmez et al. [53] and Kim et al. [54], who showed no statistically significant effect on the FS of Vita Enamic after aging.

The high occlusal load affects mainly posterior teeth; therefore, deformation or fracture of restorative material might occur in such restorations. It is worth emphasizing that a lower elastic modulus indicates that a material is characterized by low stiffness, and thus greater visco-elasticity; this latter property is not appropriate for restorative materials advocated for posterior restorations [37].

In the present study, Grandio and Vita showed the highest modulus of elasticity before (16.3 GPa and 16.9 GPa, respectively) and after aging (19.4 GPa and 18.9 GPa, respectively). Most studies showed no significant relationship between the aging of materials and changes in the modulus of elasticity [40,45,52]; this is in accordance with the results of the current study.

Some studies established that there may be a correlation between elastic modulus and filler volume; the higher the filler content, the greater the flexural modulus [41,55]. This concept seems to support the results of our current study. It is important to mention that the modulus of elasticity of both Grandio and Vita corresponds to the values of sound human dentin (8.7–30 GPa) [45,56]. On the other hand, all materials differed from the flexural modulus of enamel (50–120 GPa) [39,41].

The evaluation of the water sorption of the tested materials was performed in this study according to the ISO 4049:2009 specification; polymer-based restorative materials should exhibit a maximum sorption up to 40 µg/mm^3^ [57]. All tested CAD/CAM materials met the ISO standard requirements for water sorption. However, it is well known that excessive water sorption by restorative materials can cause degradation of the material due to softening of the resin matrix and to elution of monomers and other degraded products.

In the present study, the specimens were immersed in distilled water. However, different storage media, such as water [58], artificial saliva [59], 75% ethanol/water [47], ethanol [60], and sodium chloride [61], have been employed in previous investigations to evaluate sorption evaluation and to simulate some conditions of the oral cavity. Several studies showed that the type of medium and storage time can affect the degree of sorption [47,58,62]. Moreover, storage in ethanol showed a significant increase in sorption compared to aqueous solutions [47,62]. Red wine, acidic drinks, coffee, curry, or cress solutions were also used to assess the colour change in restorative materials [63,64]. Additionally, some studies showed the negative effects that different beverages can have on the elution of monomers [21,65].

It has been advocated that, with the increase of filler, the content of the polymer matrix decreases, which reduces the diffusion of water [14,58,66]. This was in accordance with the results of the current study; the material with the lowest percentage content of filler (Shofu) exhibited a significantly higher water sorption compared to the other materials (*p* < 0.05). Conversely, similar results of water sorption were obtained for Vita and Grandio in the current study. This outcome may suggest that the water sorption is more influenced by the composition (filler and resin content) rather than the manufacturing technique itself [58].

An increase in water sorption may also depend on the polymer matrix composition of the tested materials. For instance, TEGDMA has been demonstrated to have the highest water sorption, followed by Bis-GMA, UDMA, and Bis-EMA [19,58]. Alamoush et al. [2] reported that the Lava Ultimate (resin composite blocks) containing all the above-mentioned resin monomers exhibited highest water sorption [58]. The results of the present study did not confirm such a relationship because Vita and Shofu contain the same monomers (UDMA, TEGDMA) and differed significantly in terms of water sorption (5.4 µg/mm^3^ and 22.8 µg/mm^3^, respectively). Therefore, it might be hypothesized that water sorption might depend on the percentage content of the polymer matrix, although the monomers of Grandio are not disclosed by the manufacturer.

Hardness can be related to the ability of a material to resist permanent surface indentations; it characterizes the material’s wear and scratching resistance [5]. The materials used in restorative dentistry replace missing dental tissue, and thus should exhibit a hardness at least similar to that of enamel and/or dentine.

Vita showed the highest value of hardness (>200 KHN) between the three materials tested in this study, suggesting that it may be an ideal material for enamel replacement [5,39]. In general, hardness reduction of polymer-based materials subsequent to aging is greater than that of hybrid-ceramic materials; this is mainly due to the penetration of solvents into the matrix, which causes resin plasticization and a softening effect [2,63]. Studies showed a negative correlation between filler amount and hardness reduction over time. In other words, it seems that the higher the filler content, the smaller the decrease in hardness [2,67,68,69]. This is in accordance with the present study, where the two resin-based materials (Grandio disc multicolor and Shofu Block HC) showed a significant reduction in microhardness after aging (*p* < 0.05). Moreover, Vita exhibited the highest surface hardness before and after storage (*p* < 0.05). Shofu with the highest percentage of polymer matrix showed the greatest softening ratio (25.4%).

Materials can have different reductions in hardness depending on the storage and time of storage. Indeed, solutions such as coffee or acidic beverages cause a reduction in microhardness of CAD/CAM composite materials [63,70].The hardness of the tested materials decreased after ethanol storage in accordance with the results reported in previous studies [71,72].

Finishing and polishing of the surface of CAD/CAM materials may influence the clinical success, as well as the longevity of the restoration. Indeed, surface roughness, voids, and outcrops may favour bacterial adhesion and plaque accumulation; as a consequence, secondary dental caries and/or periodontitis in proximity of cervical margins may occur [45,73,74]. High roughness can promote superficial cracks’ formation and increase surface wearing, thus affecting the stability of the final restoration [75,76].

Surface roughness can be assessed using profilometry, scanning electron, and atomic force microscopy [77]. The profilometer is more often used because it allows for quantitative measurement of surface characteristics [77]. Moreover, the most frequently tested parameter is the average arithmetic height (Ra) [77,78]. The mean width of the profile elements (RSm) and the average maximum height of the profile (Rz din) were also measured in our study, as well as the Ra. However, previous studies have also evaluated other roughness parameters, such as root mean square roughness (Rq), valley roughness (Rv), and peak roughness (Rp) [79].

The results of the present study showed that Ra, RSm, and Rz din increased for all of the tested materials after aging. However, Ra and RSm were significantly greater only for Shofu after ethanol storage (*p* < 0.05). In a previous study, a significant increase in the Ra of Vita Enamic was observed, but without exceeding critical Ra values (Ra > 0.2 μm) [45,80]. The values for Grandio and Vita were clinically acceptable, while Ra of Shofu exceeded the critical values reported in the literature [42]. Some authors showed an increase in roughness parameters (Ra and RSm) in hybrid ceramics such as Vita Enamic when submitted to wear simulation [81]. An increase in roughness parameters may adversely cause a faster wear of the material’s surface. It is worth highlighting that appropriate polishing procedures may reduce the values of roughness parameters, although the material composition (size, ratio, type of inorganic filler, and structure of the organic matrix) can influence the final surface roughness [74,75,77,82].

In the present study, SEM analysis specimens revealed that ethanol aging contributed to a slight increase in porosities due to the loss of the spherical fillers in the specimens of Shofu. A previous study also found microcracks (voids) between the inorganic and organic components in the aged composite specimens [72].

The limitations of the present in vitro study should be acknowledged. Firstly, the design of the study does not fully reflect oral cavity conditions. Therefore, further in vitro and clinical studies should be performed to properly assess the qualities and the limitations of the tested materials. In particular, the longevity and durability of these materials when bonded to dental substrates and submitted to in vivo occlusal stress or in vitro using dynamic loading in a chewing simulator ought to be evaluated in more complex conditions.

## 5. Conclusions

Despite the comparable modulus of elasticity of Vita Enamic multiColor and Grandio disc multicolor, the latter of these showed greater flexural strength and ultimate tensile strength both at baseline and after aging. Hence, Grandio multicolor may be potentially employed not only for anterior restorations, but also for those restorations requiring load-bearing capacity. Conversely, aging seems to affect several properties of Shofu Block HC, so its use for permanent restorations should be well-pondered based on the clinical situation.

## Figures and Tables

**Table 1:** The materials used in the study.

Material	Type	Composition		Filler Weight (w%)
		Inorganic Fillers/Structure	Main Resin Monomers	
Grandio disc multicolor [Grandio] {VOCO GmbH, Cuxhaven, Germany}	Composite resin	Barium aluminium borosilicate glass, silicon dioxide	Not disclosed by the company	86
Shofu Block HC (two-layered block) [Shofu] {Shofu Inc., Kyoto, Japan}	Composite resin	Silica, fumed silica and zirconium silicate	UDMA, TEGDMA	75
Vita Enamic multiColor (Blocks) [Vita] {Vita Zahnfabrik, Bad Sackingen, Germany}	Resin-ceramic material (PICN)	Feldspar ceramic enriched with aluminium oxide (86 wt%): SiO_2_ (58–63%), Al_2_O_3_ (20–23%), Na_2_O (9–11%), K_2_O (4–6%), B_2_O_3_ (0.5–2%), ZrO_2_ (<1%), KaO (<1%)	UDMA + TEGDMA (14 wt%)	86

Material description and composition are presented as per manufacturers’ information. TEGDMA = triethylenglycol dimethacrylate; UDMA = urethane dimethacrylate; PICN = polymer-infiltrated-ceramic network. [abbreviations]; {manufacturers}.

**Table 2:** Distribution of elastic modulus (GPa), ultimate flexural strength (MPa), and ultimate tensile strength (MPa) among the experimental groups and evaluation times.

	UTS (MPa)		FS (MPa)		Modulus of Elasticity (GPa)	
Block	Baseline	After Aging	Baseline	After Aging	Baseline	After Aging
Grandio disc multicolor	164.5 ± 17.6 ^A,a^	159.6 ± 17.7 ^A,a^	324.7 ± 7.45 ^A,a^	342.4 ± 28.1 ^A,a^	16.3 ± 1.31 ^A,a^	16.9 ± 0.73 ^A,a^
Shofu Block HC	123.8 ± 16 ^A,b^	68.9 ± 17.9 ^B,c^	164.3 ± 9.72 ^A,b^	173.1 ± 4.90 ^A,b^	10.9 ± 0.99 ^A,b^	8.8 ± 0.60 ^B,b^
Vita Enamic multiColor	108.4 ± 15.6 ^A,b^	102.6 ±17.2 ^A,b^	161.1 ± 11.7 ^A,b^	165.1 ±10.6 ^A,b^	19.4 ± 1.54 ^A,a^	18.9 ±1.33 ^A,a^

Different capital letters indicate statistically significant differences in rows (baseline vs. aging). Different lower-case letters indicate statistically significant changes in columns (baseline or aging). Two-factor ANOVA repeated measures were followed by a Tukey post-hoc test (*p* < 0.05).

**Table 3:** Distribution of sorption (µg/mm^3^) between the experimental groups of composite blocks.

Block	Sorption (µg/mm^3^)
Grandio disc multicolor	5.70 ± 0.68 ^a^
Shofu Block HC	22.8 ± 3.37 ^b^
Vita Enamic multiColor	5.44 ± 0.45 ^a^

Different lower-case letters indicate statistically significant differences among the rows (*p* < 0.05). One-factor ANOVA (Welch) was followed by Games–Howell post-hoc test.

**Table 4:** Distribution of microhardness (KHN) among the experimental groups and evaluation times. Softening ratio is calculated as the percentage difference between values after aging.

Block	Microhardness (KHN)		Softening Ratio
	Baseline	After Aging	
Grandio disc multicolor	163.1 ± 4.9 ^A,a^	153.8 ± 6.2 ^B,a^	5.6%
Shofu Block HC	72.8 ± 2.2 ^A,b^	54.3 ± 3.3 ^B,b^	25.4%
Vita Enamic multiColor	211.8 ± 4.5 ^A,c^	208.5 ± 6.4 ^A,c^	1.6%

Different capital letters indicate statistically significant differences in rows (baseline vs. aging). Different lower-case letters indicate statistically significant changes in columns (baseline or aging). Two-factor ANOVA repeated measures were followed by a Tukey post-hoc test (*p* < 0.05).

**Table 5:** Distribution of roughness parameters among the experimental groups and evaluation times.

Block	Ra (µm)		RSm (mm)		Rz Din (µm)	
	Baseline	After Aging	Baseline	After Aging	Baseline	After Aging
Grandio disc multicolor	0.077 ± 0.017 ^A,a^	0.086 ± 0.020 ^A,a^	0.053 ± 0.018 ^A,a^	0.059 ± 0.014 ^A,a^	0.769 ± 0.166 ^A,a^	0.786 ± 0.124 ^A,a^
Shofu Block HC	0.305 ± 0.059 ^A,b^	0.327 ± 0.060 ^B,b^	0.096 ± 0.033 ^A,b^	0.103 ± 0.032 ^B,b^	3.59 ± 0.464 ^A,b^	3.68 ± 0.636 ^A,b^
Vita Enamic multiColor	0.199 ± 0.031 ^A,c^	0.202 ± 0.027 ^A,c^	0.036 ± 0.003 ^A,c^	0.037 ± 0.004 ^A,c^	1.61 ± 0.221 ^A,c^	1.630 ± 0.223 ^A,c^

Different capital letters indicate statistically significant differences in rows (Baseline vs. aging). Different lower-case letters indicate statistically significant changes in columns (baseline or aging). Two-factor ANOVA repeated measures were followed by a Tukey post-hoc test (*p* < 0.05).

## Data Availability

Data are available on request to the corresponding author.
